# HIFU for the treatment of gastric cancer with liver metastases with unsuitable indications for hepatectomy and radiofrequency ablation: a prospective and propensity score-matched study

**DOI:** 10.1186/s12893-021-01307-y

**Published:** 2021-07-12

**Authors:** Bin Zhou, Ning He, Jiaze Hong, Tong Yang, Derry Minyao Ng, Xudong Gao, Kun Yan, Xiaoxiang Fan, Zhi Zheng, Ping Chen, Jianjun Zheng, Qi Zheng

**Affiliations:** 1 Department of General Surgery, HwaMei Hospital, University of Chinese Academy of Sciences, Northwest Street 41, Haishu District, Ningbo, 315010 Zhejiang China; 2 Ningbo Institute of Life and Health Industry, University of Chinese Academy of Sciences, Ningbo, Zhejiang China; 3 Key Laboratory of Diagnosis and Treatment of Digestive System Tumors of Zhejiang Province, Ningbo, Zhejiang China; 4 Department of Tumor HIFU Therapy, HwaMei Hospital, University of Chinese Academy of Sciences, Ningbo, Zhejiang China; 5 The Second Clinical Medical College, Zhejiang Chinese Medical University, Hangzhou, Zhejiang China; 6 Medical College of Ningbo University, Ningbo, Zhejiang China; 7 Department of Gynecology, HwaMei Hospital, University of Chinese Academy of Sciences, Ningbo, Zhejiang China; 8 Department of Medical Image, HwaMei Hospital, University of Chinese Academy of Sciences, Ningbo, Zhejiang China; 9 Department of Interventional Therapy, HwaMei Hospital, University of Chinese Academy of Sciences, Ningbo, Zhejiang China

**Keywords:** High intensity focused ultrasound, Gastric cancer with liver metastases, Palliative chemotherapy, Best supportive care, Prognosis

## Abstract

**Background:**

The purpose of this study was to explore the efficacy and safety of high intensity focused ultrasound (HIFU) in gastric cancer with liver metastasis (GCLM) patients who were contraindicated for either hepatectomy or radiofrequency ablation (RFA).

**Methods:**

This is a prospective, observational study on GCLM patients with 1–3 liver metastases. The primary gastric lesions were thoroughly resected and any case that exhibited extra-hepatic metastasis was excluded. A 1:2:2 propensity score-matching analysis was performed using a logistic regression model on the HIFU group, best supportive care (BSC) group, and palliative chemotherapy (PC) group. The primary endpoints include progression-free survival (PFS) and overall survival (OS).

**Results:**

Forty patients were finally included, there were 8 cases in HIFU group, 16 cases in BSC group, and 16 cases in PC group. The median follow-up time for the entire cohort was 10 months. The median PFS was 16.5 months in HIFU group, 2 months in BSC group, and 5 months in PC group. The median OS was 27.5 months in the HIFU group, 7 months in the BSC group, and 11.5 months in the PC group. Additionally, no grade 3 or higher adverse events occurred in the HIFU group.

**Conclusion:**

The results of this study showed that HIFU treatment could improve the long-term prognosis of GCLM patients without a significant increase in the occurrence of adverse events. Compared with PC and BSC, HIFU is the preferred treatment option when GCLM patients without extra-hepatic metastasis are unable to undergo either surgery or RFA.

**Supplementary Information:**

The online version contains supplementary material available at 10.1186/s12893-021-01307-y.

## Introduction

Gastric cancer (GC) is the fifth most commonly diagnosed malignancy and the third leading cause of death from cancer worldwide [1]. The main reason for the high mortality rate is that the prognosis of advanced GC is usually poor, especially in patients with distant metastasis [2]. The liver is one of the most common metastatic sites from GC. About 10% of patients with GC are initially diagnosed with liver metastasis and liver metastases occur in about 37% of patients after radical gastrectomy [3, 4]. Previous studies have shown that systemic chemotherapy is the recommended treatment for patients with gastric cancer with liver metastasis (GCLM) [5]. However, there is growing evidence that when the metastatic lesions are limited to the liver, local treatments such as hepatectomy and radiofrequency ablation (RFA) are viable alternative therapies [6, 7]. It has been reported that the 5-year overall survival (OS) of patients with GCLM without extra-hepatic metastasis is increased by 10–30% after successful surgical resection of the liver metastasis [8, 9]. Similarly, RFA can achieve a better long-term effect in selected patients with GCLM, with a 5-year OS of 3–30% [10, 11].

Unfortunately, due to various reasons, a considerable number of patients with GCLM cannot receive surgery or RFA. Generally speaking, this group of patients can only receive palliative chemotherapy (PC) or best supportive care (BSC), and either choice has a poor prognosis. The median survival time of these patients is typically less than a year [12, 13].

In recent years, a new local treatment technique, high-intensity focused ultrasound (HIFU), has been used for the treatment of liver tumors, which includes both primary and metastatic liver cancers. During HIFU treatment, ultrasound waves are focused and hyperthermia up to 60–100 °C is induced in the targeted lesion. Within this temperature range, the water within the tissues begin to vaporize and microbubble formation happens after. Cavitation then ensures and cell death occurs through the process of coagulative necrosis [14, 15]. Previous studies have shown that HIFU treatment could treat, including but not limited to liver tumors and pancreatic cancer, and is associated with fewer serious adverse reactions [16, 17]. According to our previous studies, HIFU treatment for primary liver cancer and colorectal liver metastases has achieved surprising positive results [18, 19]. Therefore, we designed a prospective, propensity score matching study for patients with GCLM without evidence of extrahepatic metastases who were unable to undergo hepatectomy or RFA.

## Methods and materials

### Study design

This was a single-institution, prospective observational study to evaluate the efficacy and safety of HIFU treatment compared with BSC and PC for patients with GCLM. The database comprises data collected from GC patients from January 2014 to December 2019 at HwaMei Hospital, University of Chinese Academy of Sciences. This study was approved by the research ethics committee of HwaMei Hospital, University of Chinese Academy of Sciences (approval NO. PJ-NBEY-KY-2019-153-01). Written consent was obtained from all patients before enrollment. Patients were eligible for the study if they had liver metastases (synchronous liver metastasis, metachronous liver metastasis) from GC, and their primary lesions (gastric lesions) had been removed without evidence of extrahepatic metastasis. Patients were evaluated by HwaMei Hospital, University of Chinese Academy of Sciences multiple disciplinary team (MDT), which comprises gastrointestinal surgeons, hepatobiliary surgeons, medical oncologists, interventional radiologists, hepatologists, and HIFU oncologists. Unsuitable indications of resection include the following: determined unresectable by MDT, resectable but is considered a major or difficult surgery for patients who are in poor condition and patients who refused surgery. Metastases were judged as unsuitable for RFA due to their proximity to vessels, bile ducts, the gastrointestinal tract or gallbladder, the diaphragm, or large size (> 3 cm), etc.

Exclusion criteria: (1) The patient refused to participate in the study. (2) The pathology of gastric lesions was not gastric adenocarcinoma or the liver lesions were the primary liver cancer. (3) The number of liver metastases was more than three. (4) The patient was lost to follow-up, or the data were incomplete. Other key eligibility criteria include an Eastern Cooperative Oncology Group (ECOG) performance score of ≤ 2, adequate organ and bone marrow function, defined as having a white blood cell count > 3.5 × 10^3^/uL, neutrophil count > 1500/uL, hemoglobin > 10 g/dL, creatinine ≤ 2 × upper limit of normal (ULN), creatinine clearance 60 mL/min/1.73 m, alanine aminotransferase (ALT) or aspartate aminotransferase (AST) < 2 × ULN, bilirubin ≤ 1.5 × ULN, and platelets ≥ 100 × 10^9^/L. The additional exclusion criteria for this study are as follows: congestive heart failure, unstable angina, active cardiomyopathy, unstable ventricular arrhythmia, previous stroke < 12 months, human immunodeficiency virus, and other active malignancies. The primary endpoints include progression-free survival (PFS) and OS.

Patients were classified according to the treatment received for their liver metastases. The first group was treated with HIFU (HIFU group), regardless of prior treatment with or without other local treatments (such as chemotherapy, targeted therapy, RFA, transcatheter arterial chemoembolization (TACE), immunotherapy, etc.) for liver metastases, or continued with other treatments after the completion of HIFU treatment. The second group received BSC for liver metastases without receiving any other anti-tumor treatments (BSC group), but there was no restriction on receiving adjuvant chemotherapy after radical gastrectomy was performed. Reasons for patients in the BSC group to refuse any anti-tumor therapy included, but were not limited to, the high cost of treatment, lack of confidence in treatment, intolerance or fear of treatment side effects. The third group only received PC for liver metastases (PC group) and did not receive additional relevant anti-tumor therapy (such as targeted therapy, RFA, TACE, immunotherapy, etc.). The chemotherapy regimen used is based on 5-fluorouracil (5-FU) or platinum.

### HIFU treatment

The JC200 + HIFU system (Chongqing Haifu Technology, Chongqing, China) was used for this research. In summary, it has a transducer with a diameter of 20 cm, a focal length of 16.5 cm, and an operating frequency of 1.0 MHz with a focal region of 3 × 3 × 8 mm. Refer to the previous study for operational details [18]. A treatment plan was devised with a 3-dimensional reconstruction of the tumor boundary with 5-mm-separated sections. Typically, the planned treatment volume should exceed the tumors’ margin by at least 5 mm.

Systemic therapy can be continued one week after the operation according to the needs of the disease. The adjuvant therapy regimen for all patients was determined by the oncologist or MDT.

### Follow-up

Adverse events (AEs) were graded using the National Cancer Institute Common Terminology Criteria for Adverse Events (CTCAE), version 4.0 [20]. In the HIFU group, the tumor responses were assessed using the modified Response Evaluation Criteria in Solid Tumors (mRECIST) [21]. Complete response (CR): the disappearance of any intratumoral arterial enhancement in all target lesions. Partial response (PR): at least a 30% decrease in the sum of diameters of viable (contrast enhancement in the arterial phase) target lesions, taking as reference the baseline sum of the diameters of target lesions. Progressive disease (PD): an increase of at least 20% in the sum of the diameters of viable (enhancing) target lesions, taking as reference the smallest sum of the diameters of viable (enhancing) target lesions that were recorded just prior to the initiation of the treatment. Stable disease (SD): any cases that do not qualify for either PR or PD. Tumor response was assessed on contrast-enhanced MRI or CT and was carried out on day 30 and every 3 months thereafter.

In both the BSC and PC groups, tumor response was determined according to the Response Evaluation Criteria in Solid Tumors (RECIST) [22]. PFS is defined as the time from the first HIFU treatment/BSC/PC to death, locoregional recurrence, or distant recurrence. OS is defined as the time from completion to the first HIFU/BSC/PC to death from any cause or lost follow-up. The median follow-up time for the entire cohort was 10 months (range 3–44 months), and follow-up of all patients included in this study was stopped on December 2020.

### Statistical analysis

To reduce selection bias, a 1:2:2 propensity score matching analysis was performed on the HIFU group, BSC group, and PC group. Propensity scores were estimated using a logistic regression model and the following covariates: age, gender, body mass index (BMI), ECOG, Child–Pugh class, the time of liver metastases, the number and size of liver lesions, TNM stage of GC, the number of lymph nodes retrieved. A caliper equal to 0.20 of the standard deviation of the logit of the propensity score was used to prevent poor matches. Using these propensity scores, patients with HIFU treatment (HIFU group) were individually matched to patients with BSC (BSC group) and patients with PC (PC group) [23].

Hazard ratios (HR) and 95% confidence intervals (CI) were calculated. Time to progression and survival was evaluated using the Kaplan–Meier method. PFS was calculated using the Kaplan–Meier method, and the log-rank test was employed to determine the significance. Continuous variables were compared using the independent-samples t-test or Wilcoxon rank-sum test, and categorical variables were compared using Pearson’s chi-squared test or Fisher’s exact test, when appropriate. All statistical tests were performed 2-sided, and *p* < 0.05 was considered statistically significant. Analyses were performed using SPSS software (version 25.0, SPSS Inc. IL, USA).

## Results

A total of 960 GC patients underwent gastrectomy at HwaMei Hospital, University of Chinese Academy of Sciences between January 2014 and December 2019, of which 331 patients were diagnosed with liver metastasis from GC during treatment, and were follow-up with during this study. After the selection process was performed based on the above-mentioned inclusion and exclusion criteria, a total of 172 GCLM patients initially met the conditions of this study, there were 8 patients in the HIFU group, 55 patients in the BSC group, and 109 patients in the PC group.

There was no difference in age between the patients in the PC group and the HIFU group, but patients in the BSC group were older, with a statistically significant difference. There was no significant difference in gender, comorbidity, Child–Pugh class, the time of liver metastases, and the number of lymph nodes retrieved among the three groups. In terms of BMI, there was no difference between the PC group and the HIFU group, and patients in the BSC group had a lower BMI. Patients with the HIFU had the best ECOG, followed by the PC group and the BSC group, with statistically significant differences. The proportion of patients receiving preoperative chemotherapy was lower in the HIFU group, and higher in the PC and BSC groups. In terms of TNM stage of GC, patients in the BSC group had most advanced tumors, followed by the PC group, and the HIFU group. The most number of liver lesions was found in the BSC group, followed by the PC group, and the lowest in the HIFU group. The largest mean size of liver lesions was found in the PC group and the BSC group, while the HIFU treatment group had smaller tumor diameter.

**Fig. 1:**
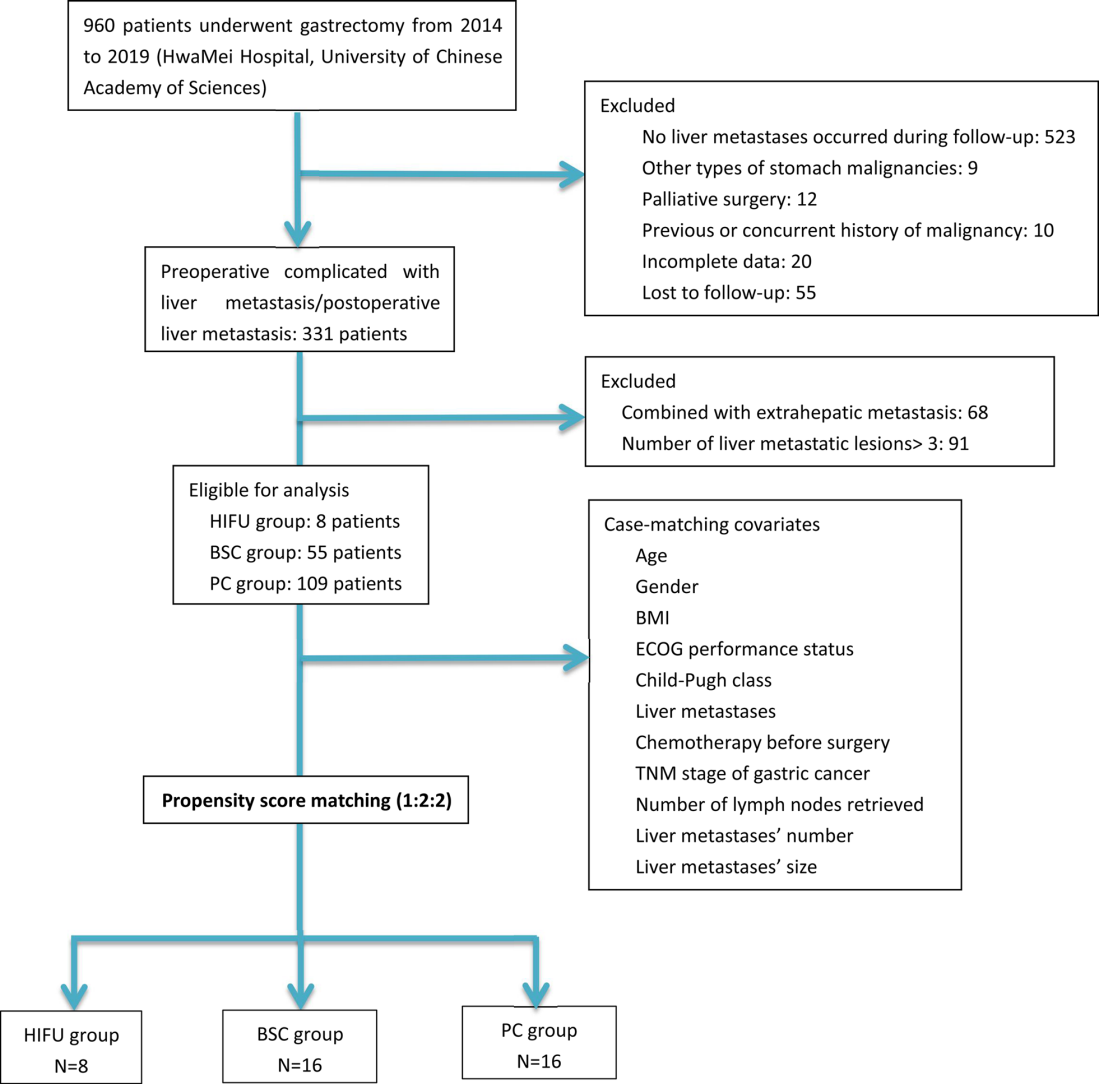
Flowchart of the study population

After performing a 1:2:2 propensity matching analysis, 40 patients were finally selected, there were 8 patients in the HIFU group, 16 patients in the BSC group, and 16 patients in the PC group. The entire flowchart is described in Fig. 1. All 40 patients (70 lesions) were unable to receive surgery or RFA, please refer to Additional file 1: Table S1 for the specific reasons. Among the three groups, it was found that there was no statistically significant difference in most of the covariates as detailed in Tables 1, 2.

**Table 1:** Basic information of the patients

Characteristic	HIFU (N = 8)	BSC (N = 16)	PC (N = 16)	p value
Age (years), Median (IQR)	61.5 (58.2–65.3)	63.2 (59.4–65.5)	62.5 (58.1–66.3)	> 0.1
Gender				> 0.1
Male	6	8	6	
Female	2	8	10	
BMI(kg/m^2^), Median (IQR)	20.5 (18.1–22.5)	19.5 (17.8–21.8)	20.0 (19.2–23.0)	> 0.1
Comorbidity				> 0.1
No	2	7	8	
Yes	6	9	8	
ECOG performance status				> 0.1
0	4	4	6	
1	4	10	9	
2	0	2	1	
Child–Pugh class				> 0.1
Grade A	7	13	11	
Grade B	1	3	5	
Liver metastases				> 0.1
Synchronous	2	1	1	
Metachronous	6	15	15	
First-line treatment of liver metastases				NA
Chemotherapy	2	–	16	
RFA	2	–	0	
HIFU + TACE	2	–	0	
HIFU	2	–	0	
Chemotherapy before surgery				0.09
No	6	16	15	
Yes	2	0	1	
TNM stage of GC				> 0.1
I	1	4	2	
II	1	3	4	
III	4	8	9	
IV	2	1	1	
Number of lymph nodes retrieved, Median (IQR)	19 (12–26)	18 (12–27)	20 (13–25)	> 0.1

*HIFU* high intensity focused ultrasound, *BSC* best supportive care, *PC* palliative chemotherapy, *BMI* body mass index, *ECOG* Eastern Cooperative Oncology Group, *RFA* radiofrequency ablation, *TACE* transcatheter arterial chemoembolization, *GC* gastric cancer, *NA* not available

**Table 2:** Liver metastases’ characteristic

Characteristic	HIFU (N = 8)	BSC (N = 16)	PC (N = 16)	p value
Previous lines of liver metastases therapy				NA
Nil	4	16	16	
1	3	0	0	
2	1	0	0	
Time from initial diagnosis to HIFU (month), (range)	0–7	–	–	
Time between last liver-directed therapy and HIFU (month), (range)	0–5	–	–	
Number				> 0.1
1	5	8	8	
2	1	4	3	
3	2	4	5	
Maximum size (cm), Median (range)	2.0 (1.2–8.8)	3.8 (1.0–8.0)	3.0 (1.0–7.0)	> 0.1
Location (liver segment)				NA
I	0	2	1	
II	1	1	3	
III	1	1	2	
IV	1	5	1	
V	1	3	4	
VI	4	7	6	
VII	1	1	3	
VIII	2	2	4	
Systematic treatment after HIFU				
No	1	–	–	
Yes	7	–	–	
Input energy of HIFU (kJ), Median(IQR)	625 (353–848)	–	–	
Input power of HIFU (W), Median(IQR)	340 (297–360)	–	–	
Cumulative transmit time of HIFU (s), Median(IQR)	1890 (1230–2219)	–	–	

*HIFU* high intensity focused ultrasound, *BSC* best supportive care, *PC* palliative chemotherapy, *NA* not available

Among the 40 patients, there was an equal proportion of males and females. The vast majority (92.5%) of patients had an ECOG of 0–1, and only three had an ECOG of 2. All participants had a liver function of Child–Pugh A or B, with 77.5% of patients having a liver function of Child–Pugh A. Liver metastases were present at the initial primary diagnosis in 10% of patients (four cases). Although there was no statically significant difference among the three groups, the rate of synchronous liver metastasis in the HIFU group was higher than that of the other two groups (25%, 6.25%, 6.25%, respectively). Three patients (2 in the HIFU group and 1 in the PC group) received chemotherapy before surgery. The post-surgery pathological staging of GC showed that 17.5% of patients were in stage I, 72.5% were in stage II-III, and 10% were in stage IV, the latter presented with liver metastasis only. Details are described in Table 1.

In the HIFU group, half of the patients received line 1 or above treatment. The other two groups of patients did not receive any additional anti-tumor therapy due to liver metastasis. Approximately half of the patients had a single lesion, eight had two lesions and 11 had three lesions. Most patients (87.5%) received systematic treatment after HIFU was performed. More details are described in Table 2. All patients successfully underwent HIFU treatment without any complications during the operation.

### Long-term outcome

**Fig. 2:**
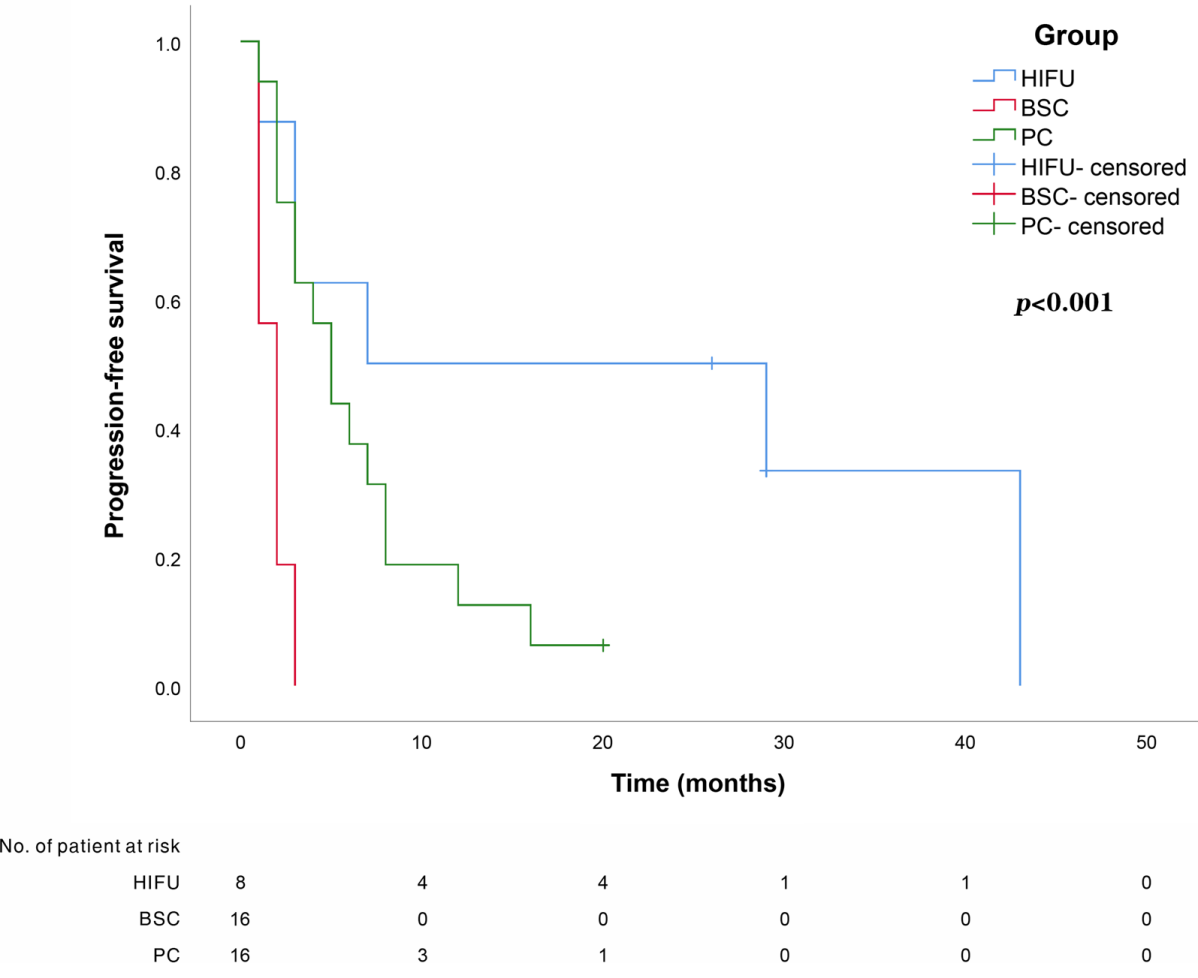
Comparison of survival curves for progression-free survival (PFS) among HIFU group, BSC group, and PC group (*p* < 0.001)

**Fig. 3:**
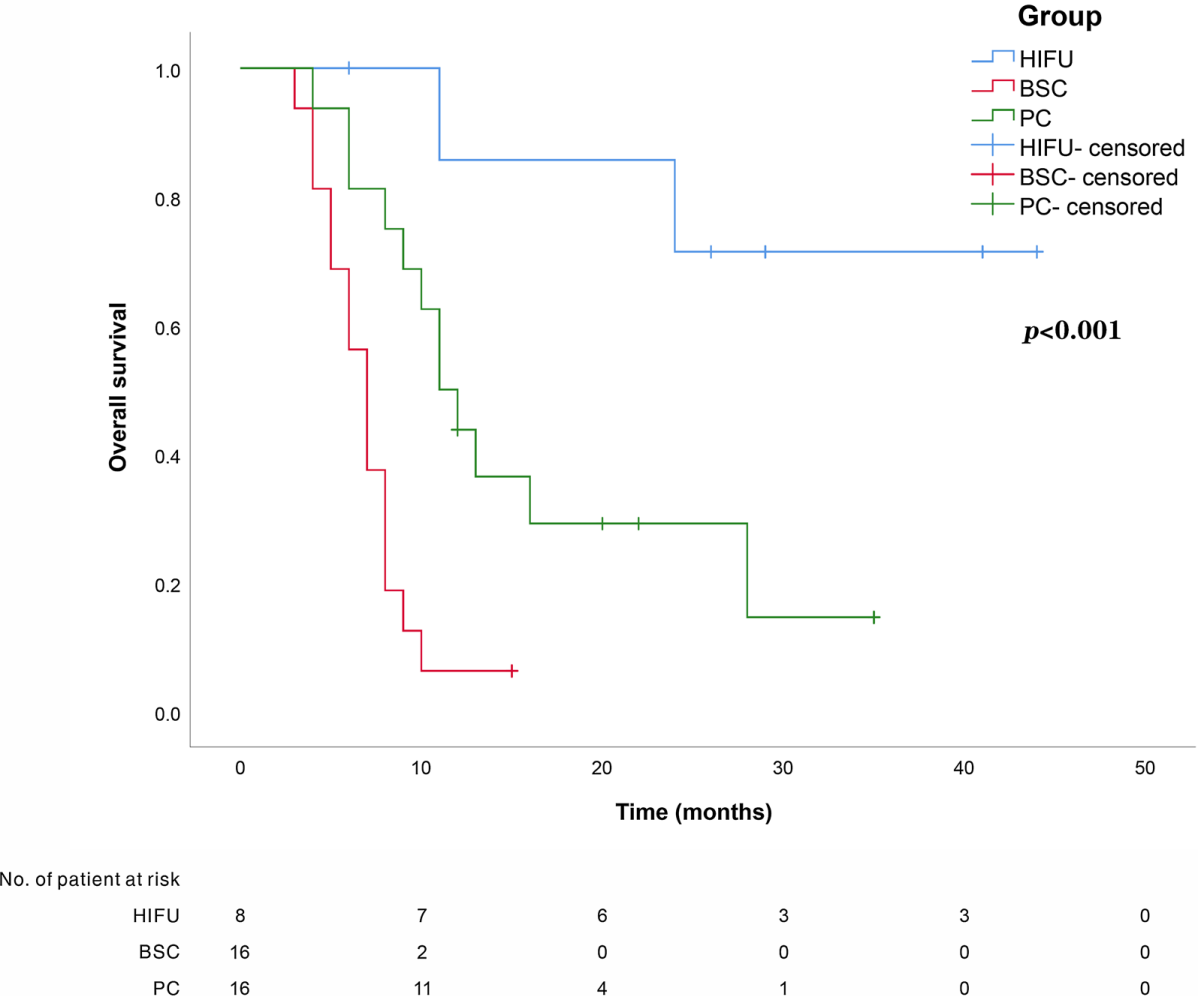
Comparison of survival curves for overall survival (OS) among HIFU group, BSC group, and PC group (*p* < 0.001)

The median PFS was 16.5 months in the HIFU group, 2 months in the BSC group, and 5 months in the PC group. Kaplan–Meier curves were used to determine the PFS of the HIFU group, BSC group, and PC group. The PFS of the HIFU group was significantly higher than that of the other two groups, while the PFS of the PC group was significantly higher than that of the BSC group (Fig. 2). The median OS was 27.5 months in the HIFU group, 7 months in the BSC group, and 11.5 months in the PC group. Among the three groups, the patients who received HIFU had the best OS, followed by the patients who received PC, and the patients who received BSC had the worst OS (Fig. 3). At the end of the follow-up period, 11 patients (27.5%) were still alive, of whom 3 had no evidence of recurrence (2 in the HIFU group, 1 in the PC group) and 29 died(72.5%) from tumor progression. No patients were lost to follow-up.

### Tumor response

Evaluation of therapeutic response with contrast-enhanced MRI was performed 30 days after the HIFU treatment. All lesions (a total of 13) were suitable for assessment. Five patients (62.5%) achieved CR and two achieved PR, the remaining patient achieved SD. The objective response rate (ORR) was 87.5%. At the time of data analysis, two patients were alive at 26 and 29 months without any evidence of recurrence. Four developed liver recurrence or distant metastasis and were alive. Two patients with recurrence died due to tumor progression (Table 3).

**Table 3:** Patient’s details

No	Gender	Age	ECOG	Previous lines of liver metastases therapy	HIFU treatment	Tumor response^a^	Systematic treatment after HIFU	Recurrence after first HIFU	Outcome (month)
1	Male	71	0	–	HIFU	PR	Tegafur	Hepatic	Expired (24)
2	Female	67	1	–	HIFU	CR	Tegafur	Extrahepatic	Alive with recurrence (44)
3	Male	62	0	–	TACE + HIFU	CR	–	Hepatic	Alive with recurrence (44)
4	Female	55	1	RFA	HIFU	CR	Paclitaxel + Tegafur	Extrahepatic	Alive with recurrence (41)
5	Male	62	1	RFA; TACE	HIFU	CR	Tegafur	–	Alive without recurrence (29)
6	Male	71	0	SOX	HIFU	CR	SOX	–	Alive without recurrence (26)
7	Male	64	1	–	TACE + HIFU	SD	Nab-paclitaxel + Tegafur	Extrahepatic	Expired (11)
8	Male	59	0	SOX	HIFU	PR	–	Hepatic	Alive with recurrence (6)

*ECOG* Eastern Cooperative Oncology Group, *HIFU* high intensity focused ultrasound, *RFA* radiofrequency ablation, *TACE* transcatheter arterial chemoembolization, *SOX* oxaliplatin and tegafur, *CR* complete response, *PR* partial response, *SD* stable disease

^a^Contrast-enhanced MRI or CT was performed at 30 days after HIFU treatment

In the BSC group, all patients exhibited tumor progression, only one patient was alive at 15 months, and the remaining 15 patients died from tumor progression. In the PC group, 3 patients achieved CR, 6 patients achieved PR, 3 patients achieved SD and PD occurred in 4 patients. The ORR was 56.3%. One patient was alive at 20 months without any evidence of recurrent disease, and three patients developed either liver recurrence or distant metastasis and were alive. The remaining 12 patients died from tumor progression.

### Treatment-related AEs

Acute toxicities that occurred during the HIFU treatment period and for 30 days after completion of the treatment are described in this section. Any grade AEs were observed in four cases. Pain and fatigue were the most common (n = 3). Others include fever, increased ALT/AST, and skin edema. Notably, no grade 3 or higher AEs occurred. Table 4 summarized all AEs related to the HIFU treatment.

**Table 4:** All any grade adverse events (AEs) related to HIFU treatment after 30 days

Toxicity	Grade 1	%	Grade 2	%
Any adverse events	4	50.0	2	25.0
Fatigue	3	37.5	0	–
Pain	3	37.5	2	25.0
Fever	2	25.0	0	–
Skin edema	1	12.5	0	–
ALT/AST increased	2	25.0	1	12.5

*ALT* alamine aminotransferase, *AST* aspartate aminotransferase

No Grade 3 or worse toxicities

In PC, the incidence of any grade AEs was 62.5% (10/16), and the most common AEs were nausea/vomiting, pain, and fatigue. Other AEs including diarrhea, constipation, peripheral neuropathy, anemia, leukopenia, increased ALT/AST, neutropenia, and thrombocytopenia were also observed. Grade 3–4 AEs accounted for 25% (4/16), including nausea/vomiting, leukopenia, neutropenia, and thrombocytopenia. No treatment-related deaths were observed.

## Discussion

According to the National Comprehensive Cancer Network guidelines, localized thermal treatment options such as RFA or microwave ablation for liver metastasis of GC are not considered the best supportive treatment option [24]. Systemic chemotherapy is currently the preferred treatment for patients with GCLM. There is no doubt that systemic chemotherapy can improve long-term survival and prolong PFS compared with BSC [25, 26]. TACE and hepatic arterial infusion chemotherapy have also been reported to be used for the treatment of GCLM, but only in small sample retrospective analysis and case reports [27–29]. At present, some scholars have proposed that patients with GCLM can be divided into three categories: potentially resectable tumor (category I), marginally resectable tumor (category II), and unresectable tumor (category III), this determination should be made by an MDT [6, 30]. There is also another group of scholars who were inspired by the evidence of substantially increased survival benefits observed in patients who received surgery for colorectal liver metastases and begun to further explore the role of surgery for GCLM [31].

Palliative gastrectomy is currently not recommended for patients with advanced GC (stage IV) unless perforation, bleeding, pyloric obstruction, etc. occurs [4, 32]. However, when the metastatic lesions can be removed completely, expanded surgery may improve the patients’ prognosis. A retrospective study of 28 patients with GCLM with and without extrahepatic metastasis showed a 5-year survival rate of 32% after undergoing hepatectomy, with a median OS time of 49 months [9]. Another study involving 25 GC patients with synchronous liver metastasis showed that undergoing hepatectomy resulted in a 5-year recurrence-free survival rate of 11.1% and a 5-year OS of 29.4% [8]. It should also be noted that many patients lose the chance of surgery due to either the difficulty of surgical resection or the patients’ intolerance to surgery. Fortunately for such patients, RFA can be a viable treatment option for GCLM patients who are unable to undergo hepatectomy. Oki et al. reported that a total of 94 GCLM patients received either surgery or RFA or a combination of both and that the 5-year OS and 5-year recurrence-free survival rate was 42.3% and 27.7%, respectively [33]. Two observational studies compared the short- and long-term results of RFA with liver resection for GCLM patients. The results showed that there was no difference between the 3-year and 5-year survival rates of patients undergoing either RFA and surgery and that RFA had even fewer complications [10, 11].

Unfortunately, RFA has several limitations. RFA is not able to treat lesions located near the main biliary tract or gallbladder, major blood vessels, and just beneath the diaphragm [34]. PC is usually used to treat patients with GCLM who are unable to receive either RFA or surgery, but its long-term effects are disappointing. In recent years, HIFU has been increasingly used in the treatment of liver cancer [35, 36]. Due to its characteristics, some studies including our previous studies have shown that HIFU can be used as a potential treatment option for patients with liver cancer but are contraindicated for either RFA or surgery [18, 19]. In a previous study, we reported on 13 patients with colorectal liver metastases who were contraindicated for either resection or RFA and eventually received HIFU treatment. The 2-year PFS was 16.7%, and the median PFS was 9 months. Notably, the 2-year OS was 77.8%, and the median OS time was 25 months [18]. However, the long-term prognosis of patients with either GCLM or colorectal liver metastases who underwent primary lesion resection combined with hepatectomy is significantly different. The 5-year survival rate of GCLM is about 20% [10, 37], while that of colorectal liver metastases is as high as 50% after surgery [38, 39]. More importantly, few studies have evaluated the safety and efficacy of HIFU treatment for GCLM patients. Therefore, this is the first study to date that compares the long-term effects of HIFU, PC, and BSC in GCLM patients without extra-hepatic metastasis who were contraindicated for either hepatectomy or RFA. In order to increase the credibility of the study and to reduce the heterogeneity among the treatment groups, propensity matching analysis is used.

In this study, 40 patients with GCLM were included, and the baseline comparisons among the three groups were equivalent. Relevant studies have shown that the potential prognostic factors included but were not limited to age, the number of metastases, and size of metastases [7, 40, 41]. Among them, the number of lesions was the most significant prognostic factor. Multiple studies have shown that patients with a single metastasis have a better prognosis than patients with multiple metastases. Fortunately, the three groups in this study were similar in many aspects. In terms of tumor response, due to differences in treatment methods, the patients in the HIFU group were assessed using the mRECIST, while the patients in the PC and BSC groups were evaluated according to RECIST. The results of our study showed that more than half of the patients could achieve CR after HIFU treatment, while less than one-fifth of patients achieved CR in the PC group. In the BSC group, the tumor continued to progress. Similarly, the ORR was higher in the HIFU group than in the PC group. This suggests that HIFU treatment of metastatic lesions is potentially more beneficial compared with PC and BSC. In addition, no rapid tumor progression was observed during the follow-up of the three patients who did not achieve CR.

More importantly, survival analysis showed that patients treated with HIFU had the best OS and PFS, followed by PC, while the BSC group had the worst survival rate. The median survival time in the BSC group was about half a year, which is in line with previous studies [42, 43]. The median survival time of the patients who underwent PC was about twice as long as that of the BSC group, up to approximately 1 year. Picado et al. included 3000 patients with GCLM, and the results of this analysis showed that the median survival time of patients receiving PC was about 9.7 months. The median survival time after receiving palliative gastrectomy was 15.3 months, while the median survival time after receiving combined hepatectomy was 24.3 months [40]. Zhang et al. [32] reported that the median survival time for GCLM patients receiving BSC was only 2.8 months, compared with 9.4 months for those receiving systemic chemotherapy. Additionally, patients receiving multi-line chemotherapy had a longer median survival time compared to those receiving single-line chemotherapy (14.2 months versus 6.6 months). A phase II clinical trial showed that capecitabine combined with paclitaxel resulted in a median survival time of 10.1 months for GCLM patients [44]. Therefore, systemic chemotherapy rarely prolongs the median survival time of GCLM patients by more than 2 years.

Interestingly, our research showed that the median survival time of GCLM patients who underwent HIFU treatment was more than 2 years, which was twice that of the PC group, and the median PFS time was more than 1 year, which was 3 times that of the PC group.

HIFU due to its minimal invasiveness and safety characteristics is becoming more widely used in the treatment of liver cancer by inducing precise lesion coagulation necrosis without damaging the surrounding structures. Furthermore, HIFU treatment is reported to have the ability to enhance the patients’ tumor immune response [45]. A case of gastric leiomyosarcoma with liver metastases and multiple retroperitoneal lymphatic metastases was reported to be treated with HIFU combined with tetrahydropalmatine and oxaliplatin-based transarterial chemoembolization and the PFS was 4 months after treatment [46]. Park et al. reported in 2009 on 3 GCLM patients who received HIFU treatment, which was generally safe, but the efficacy in that study was indeterminate [47]. It has been reported that a 35-year female patient with metachronous GCLM achieved CR after receiving HIFU combined with chemotherapy [48]. A recent retrospective study showed that HIFU therapy for metastatic liver cancer yielded better long-term results [49]. The one-year survival rate was 48.0%, and the median OS time was 12 months. However, this study had several shortcomings. First, this study included patients with multiple malignant tumors associated with liver metastasis, including colorectal cancer, pancreatic cancer, GC, breast cancer, gallbladder cancer, etc. Second, several patients had extrahepatic metastasis. Therefore, the effect of HIFU on GCLM is not well reflected in this study. Our study preliminarily showed that HIFU treatment has a good long-term effect on patients with GCLM.

In terms of treatment-related AEs, HIFU therapy has been shown to be safe and is usually tolerated well by patients. No patient had grade 3 or above AEs, and other AEs were manageable. After symptomatic treatment, all AEs were alleviated. The AEs associated with HIFU treatment was not significantly worse than the AEs associated with PC.

To the best of our knowledge, this is the first research that specifically compared HIFU treatment with PC and BSC for patients with GCLM by coupled cases. This study evaluated the safety and efficacy of HIFU for patients with GCLM and determined that HIFU treatment can be considered to be a good alternative when surgery or RFA is contraindicated for patients with GCLM. Patients who received HIFU treatment has better long-term outcomes compared to the PC and BSC groups, with no significant increase in AEs.

The limitations of this study are as follows. First, this is a prospective observational study with a small sample size. Although the propensity matching analysis is carried out, the reliability of the conclusion is lacking due to the small sample size. Second, the patients received systemic chemotherapy after HIFU treatment, which may have increased the perceived efficacy of the HIFU treatment. As a result, the therapeutic effect of HIFU may be overestimated. Therefore, this study needs to be further verification by a large sample randomized controlled trial.

## Conclusion

The results of this study showed that HIFU treatment could improve the long-term prognosis of GCLM patients without significantly increasing AEs. Compared with PC and BSC, HIFU is the preferred treatment option when GCLM patients without extra-hepatic metastasis are contraindicated for either surgery or RFA.

## Supplementary Information

**Additional file 1.** Detail of 70 lesions for 40 patients.

## Data Availability

The datasets supporting the conclusions of this article are included within the article.

## Abbreviations

HIFU: High intensity focused ultrasound
GC: Gastric cancer
GCLM: Gastric cancer with liver metastasis
BSC: Best supportive care
PC: Palliative chemotherapy
PFS: Progression-free survival
OS: Overall survival
RFA: Radiofrequency ablation
MDT: Multiple disciplinary team
ECOG: Eastern Cooperative Oncology Group
ALT: Alanine aminotransferase
AST: Aspartate aminotransferase
ULN: Upper limit of normal
mRECIST: Modified response evaluation criteria in solid tumors
TACE: Transcatheter arterial chemoembolization
CTCAE: Common terminology criteria for adverse events
AEs: Adverse events
CR: Complete response
PR: Partial response
PD: Progressive disease
SD: Stable disease
ORR: Objective response rate
HR: Hazard ratios
CI: Confidence intervals
BMI: Body mass index
