# Supplementary material for: HIFU for the treatment of gastric cancer with liver metastases with unsuitable indications for hepatectomy and radiofrequency ablation: a prospective and propensity score-matched study
Source: BMC Surg. 2021 Jul 12;21:308. doi: 10.1186/s12893-021-01307-y (PMC8273961; doi:10.1186/s12893-021-01307-y)
Supplement: Supplementary file 1 — Additional file 1. Detail of 70 lesions for 40 patients. [file 12893_2021_1307_MOESM1_ESM.docx]

***Original Research***

***HIFU for the treatment of gastric cancer with liver metastases with unsuitable indications for hepatectomy and radiofrequency ablation: a prospective and*** ***propensity score-matched study***

**Running title:** HIFU treatment for gastric cancer with liver metastases

**Lihu Gu^1, 2, 3^**^¶^**, Ning He^4^**^¶^**, Jiaze Hong^5¶^, Tong Yang^4^, Derry Minyao Ng^6^, Xudong Gao^7^, Kun Yan^8^, Xiaoxiang Fan^9^, Zhi Zheng^4^,** **Ping Chen^1^, Jianjun Zheng^8^, Qi Zheng^1^**

^1^ Department of General Surgery, HwaMei Hospital, University of Chinese Academy of Sciences, Ningbo, Zhejiang, China.

^2^ Ningbo Institute of Life and Health Industry, University of Chinese Academy of Sciences, Ningbo, Zhejiang, China.

^3^ Key Laboratory of Diagnosis and Treatment of Digestive System Tumors of Zhejiang Province, Ningbo, Zhejiang, China.

^4^ Department of Tumor HIFU Therapy, HwaMei Hospital, University of Chinese Academy of Sciences, Ningbo, Zhejiang, China.

^5^ The Second Clinical Medical College, Zhejiang Chinese Medical University, Hangzhou, Zhejiang, China.

^6^ Medical College of Ningbo University, Ningbo, Zhejiang, China.

^7^ Department of Gynecology, HwaMei Hospital, University of Chinese Academy of Sciences, Ningbo, Zhejiang, China.

^8^ Department of Medical Image, HwaMei Hospital, University of Chinese Academy of Sciences, Ningbo, Zhejiang, China.

^9^ Department of Interventional Therapy, HwaMei Hospital, University of Chinese Academy of Sciences, Ningbo, Zhejiang, China.

**Corresponding author:**

Qi Zheng

Department of General Surgery, HwaMei Hospital, University of Chinese Academy of Sciences, Northwest Street 41, Haishu District, Ningbo, 315010, Zhejiang, China

**Tel:** +8618767166800

**Fax:** +8618767166800

**E-mail:** hifuno2hospital@163.com

¶These authors contributed equally to this work.

**Email addresses**

Lihu Gu: gulihuyuanzhi@126.com

Ning He: 1564333161@qq.com

Jiaze Hong: 1653384834@qq.com

Tong Yang: tongyang2020@163.com

Derry Minyao Ng: derry.ng@hotmail.com

Xudong Gao: 690662542@qq.com

Kun Yan: Yankun330205@163.com

Xiaoxiang Fan: Doctor_kingcrimson@163.com

Zhi Zheng: 1060919400@qq.com

Ping Chen: nbchp@126.com

Jianjun Zheng: zhengjianjun@ucas.ac.cn

| Characteristics | N |
| --- | --- |
| Intolerant or refuse surgery | 6 |
| Major or difficult surgery | 9 |
| Hepatic hilum (first, second or third) | 13 |
| Adjacent to the diaphragm or heart | 10 |
| Adjacent to the gastrointestinal tract or gallbladder | 8 |
| Lesions >3cm, or irregular shape | 11 |
| Unable to be visualized on ultrasound or CT scan | 7 |

**Supplementary Table 1.** Detail of 70 lesions for 40 patients.
